# Emotional labor and job satisfaction among nurses: The mediating effect of nurse–patient relationship

**DOI:** 10.3389/fpsyg.2023.1094358

**Published:** 2023-06-05

**Authors:** Yi-wei Xu, Ling Fan

**Affiliations:** Department of Nursing, Shengjing Hospital of China Medical University, Shenyang, Liaoning, China

**Keywords:** emotional labor, job satisfaction, nurse–patient relationship, nurses, structural equation modeling

## Abstract

Emotional labor is considered an important part of the role in the nursing field. Previous studies have found inconsistencies between emotional labor and job satisfaction of nurses, this is due to the relationship between them being affected by other factors. However, the current nurse-patient relationship is tense and leads to an unsafe and unstable working environment for nurses. It has yet to be confirmed whether the nurse-patient relationship can be used as a mediating variable to further explain the association that exists between emotional labor and job satisfaction. Therefore, this study tested the mediating effect of the nurse-patient relationship between emotional labor and job satisfaction among Chinese nurses. A total of 496 nurses were included in the study. Data collection was from December 2021 to March 2022 using the convenience sampling method. SPSS 26.0 and AMOS 23.0 software were used to perform structural equation modeling and analyze the relationship between variables. The results showed surface acting negatively affected nurse-patient relationships and job satisfaction, contrary to deep acting and naturally felt emotions. The parallel mediation of nurse-patient trust and patient-centered nursing in the relationship between emotional labor and job satisfaction was found to be statistically significant. Our study highlighted the important mediation of nurse-patient trust and the importance of the positive effects of emotional labor. Future studies can use these findings as a reference to develop interventions.

## 1. Introduction

Nursing is the backbone of any health-care system, a profession essential to the health and wellbeing of all nations (Abou, 2017). As the largest component of the health-care workers, nurses’ professional expertise is essential to the effective functioning of health-care institutions (Eskandari et al., 2017). However, nurses are now faced with the challenge of overwork stress due to overtime and double shifts, as well as rising workplace bullying, leading to low morale, emotional exhaustion and decreased happiness (Islam and Chaudhary, 2022; Islam et al., 2022a,b,c; Ahmad et al., 2023), all of which may force nurses to feign happiness to meet organizational expectations, thus creating emotional labor (EL) (Aiken et al., 2002, 2010; Kim, 2018). EL is considered an important part of the role of the health care professional, especially in the nursing field (Mann and Mark, 2005; Zamanzadeh et al., 2013). There are three strategies for EL: surface acting (SA), deep acting (DA), and naturally felt emotions (NFE). SA refers to “putting on a mask” to display or disguise emotions (Arlie, 1983). DA is where service providers strive to change their emotions so that they truly match the expectations of the organization, and NFE allow employees to express the emotions they experience in the workplace naturally without any adjustment (Arlie, 1983; Grandey, 2000). In the process of caring for patients, nurses put themselves in the shoes of patients and their families, and then produce EL, which has an important impact on nurses’ job satisfaction, patient care quality, and nurse–patient relationship (Kim and Jang, 2018; Gou et al., 2021a,b).

The current nurse–patient relationship is tense and leads to an unsafe and unstable working environment for nurses. Due to working in a high workload, stress, and risk environment, nurse job satisfaction is low and declining, and even leads to burnout, higher turnover rates, and lower quality of care (Lu et al., 2016). However, in the face of the growing patient population and their needs, nurses are constantly asked to provide high-quality healthcare services and adjust their emotions according to the needs of the situation to make the nurse–patient communication smooth and effective (Wu et al., 2018; Liu et al., 2019; Gao et al., 2020). Under such circumstances, it is very meaningful to study the EL of nurses and their performance strategies.

Furthermore, studies have shown that SA is negatively correlated with job satisfaction (Gabriel et al., 2015; Wu et al., 2018; Yeh et al., 2020). In contrast, DA and NFE appear to be the better choices, as they show a positive correlation with performance to a large extent (Hülsheger and Schewe, 2011; Wu et al., 2018). However, some researchers found inconsistent results indicating that there was no significant correlation between nurse job satisfaction and DA (Shao et al., 2016; Yin and Wang, 2018). This due to the relationship between EL and job satisfaction being affected by other factors, the mechanism of which previous studies have failed to explain. Research is needed to confirm whether the nurse–patient relationship can be used as a mediating variable to further explain the association that exists between EL and job satisfaction.

Being aware of the different effects of EL strategies on nurses, nurse administrators may be more proactive in developing better policies to leverage nurses’ positive EL. Therefore, this study aims to draw attention to the positive effects of EL on nurses’ work, including interpersonal relationships and satisfaction.

## 2. Research framework and hypotheses

Studies show that EL of nurses was closely related to job satisfaction, which is defined as a pleasant or positive emotional state, an emotional orientation to work, and comes from nurses’ self-confidence, approval from patients and other professionals, and improvement in patient health (Font-Jimenez et al., 2020). It can be divided into exogenous job satisfaction including policies, wages, benefits, interpersonal relationships, etc. and endogenous job satisfaction including a sense of accomplishment, recognition, promotion opportunities, and personal growth, etc. (Herzberg et al., 1959; Cavanagh, 1992; Zhang et al., 2020). The conservation of resources theory suggests that people are motivated to acquire and protect valuable resources to meet expected future demands when individuals and groups are threatened by resource loss that comes primarily from role demands and the energy and effort expended to conform to those roles. Employees attempt to perform EL to cope with role demands and the effect of this expenditure of resources on employees depends on the more immediate return of the service encounter and the application of resources specific to the needs at hand (Hobfoll, 1989; Bai et al., 2021). However, a constant threat to valued resources culminates in adverse consequence, which can affect patient safety and quality of work (Hobfoll, 1989; Prapanjaroensin et al., 2017). The inconsistency between facial expressions and inner feelings caused by SA will result in the decrease and loss of resources, making it easier to develop emotional disorders and consuming more psychological resources to manage emotions, resulting in unrepaired intrinsic emotional resources and thereby lowering job satisfaction and performance (Grandey, 2000; Alvaro et al., 2010; Wang et al., 2023). In contrast, DA makes one’s true feelings consistent with the emotional expression expected by the organization by changing the cognitive understanding of emotional events. When the employee’s psychology reaches a balanced and harmonious state and even brings positive emotions, it will increase the individual’s emotional resources and increase job satisfaction (Alvaro et al., 2010). NFE refers to the expression of emotions as they are felt in an organic manner. When real emotions are expressed, the emotional resources continue to increase, and the satisfaction improves accordingly (Shao et al., 2016; Lee et al., 2021).

Through EL, nurses express empathy and compassion, resulting in extensive empathic interactions and close nurse–patient relationship (Williams, 2001). The nurse–patient relationship is a work-oriented, interpersonal, and caring relationship established by nurses with patients and their families through nursing activities that affect the patient’s psychological state, treatment, and rehabilitation (Pohlmann, 2006). Trust is a positive relationship that should be developed and maintained to bring social harmony (Fareed et al., 2022). The concept of trust is of particular interest to nursing as it has been identified as an important element in the nurse–patient relationship (Bell and Duffy, 2009). For the registered nurse and patient who have established mutual trust, patients demonstrated better adaptation and cooperation to improve health and expressed a sense of safety, and nurses, in turn, improved job accomplishment and satisfaction to provide competent and ethical care (Leslie and Lonneman, 2016). A people-centered integrated care service delivery system is proposed, aiming at improving healthcare services, enhancing quality of care and reducing costs (Ding et al., 2019). Past studies have extensively demonstrated the positive impact of patient participation, not only on patient safety, patient mental health, and clinical outcomes, but also on nurses’ job satisfaction, work engagement, and health care quality (Wang et al., 2018; Ding et al., 2019).

**Figure 1:**
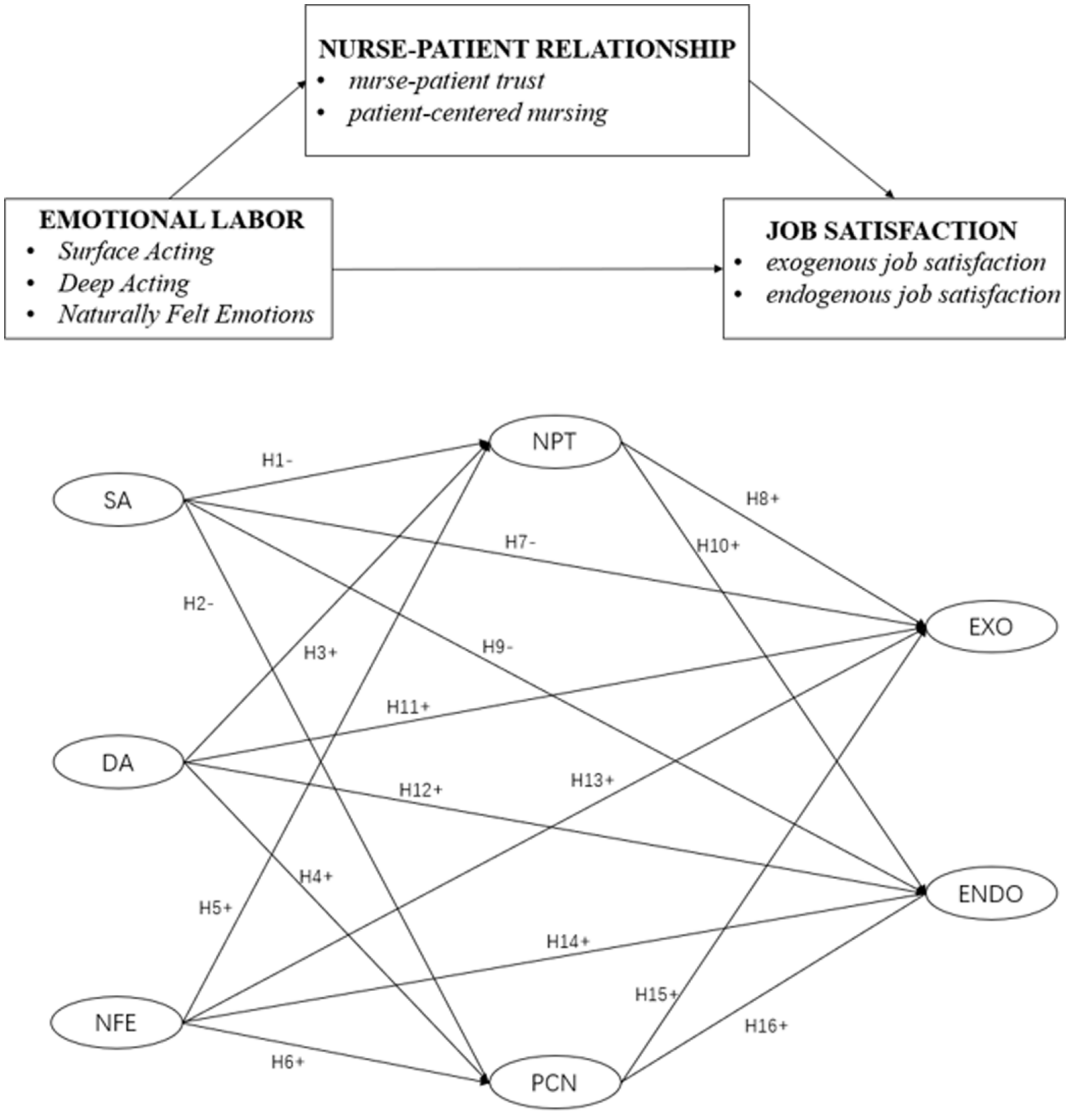
Hypothesized model of study. ENDO, endogenous job satisfaction; EXO, exogenous job satisfaction; SA, surface acting; DA, deep acting; NFE, naturally felt emotions; NPT, nurse–patient trust; PCN, patient-centered nursing.

A health-care model of EL stated that when nurses’ EL was appreciated by patients, their relationship became more harmonious, leading to nurses gaining social respect and psychological support, which was conducive to their more active involvement in medical services, and their personal identity and trust toward patients also increased accordingly (Mann and Mark, 2005). When nurses maintain the nurse–patient relationship and a caring environment, the quality of patient-centered care improves, which in turn increases job and patient satisfaction. If variable characteristics of interpersonal relationships are improved, it could effectively increase this possessive sense to the profession (Foroughi et al., 2014). In total, 16 hypothetical model paths were developed between these variables (Figure 1). Figure 1 shows the hypothesized model for this study.

## 3. Methods

### 3.1. Sample and procedure

This study included a cross-sectional survey. The convenience sampling method was used to select nurses from three hospitals in Shenyang, Liaoning Province, China, including one tertiary hospital (a large general hospital with more than 501 beds) and two secondary hospitals (regional hospitals with between 101 and 500 beds) that reflect a diversified workforce. Moreover, the study used item response theory to select a sample of 620 nurses using a standard of 20 responses for each questionnaire item (Islam et al., 2022a,b,c). The data collection time was from December 2021 to March 2022. The contents of the questionnaires for this study were entered into the online platform powered by www.wjx.cn and distributed by the heads of the nursing departments of each hospital. The inclusion criteria included (1) on-the-job, having obtained a nurse practitioner certificate; and (2) informed consent to participate in this study. The exclusion criteria included (1) those taking leave or continuing education; and (2) those who were unable to participate in this survey due to special reasons. Ultimately, a total of 496 (80% response) nurses met the inclusion criteria as study participants used in the final analysis. Among these participants, most were from tertiary hospitals (88.1%) and were female (97.6%), with an average age of 34.92 ± 5.94 years. Departments included a wide range, and internal medicine nurses (27.8%) were the most numerous. Most of the participants had worked for 11–15 years (39.9%), obtained a bachelor’s degree or above (89.3%), held the title of senior nurse (68.3%), were married (77.6%), and had an average monthly income of 6,001–9,000 RMB (47.6%).

### 3.2. Measures

The questionnaire was composed of a general information questionnaire, an emotional labor scale, a nurse–patient relationship scale, and a job satisfaction scale. The general information questionnaire included hospital level, gender, department, age, working duration, education, profession title, marital status, and income.

EL was measured using the Emotional Labor Scale, compiled by Diefendorff et al. (2005) and translated into Chinese by Bo (2006), with three dimensions and 14 items: seven items for SA, four items for DA, and three items for NFE. Each item was scored using a 5-point Likert scale ranging from 1 to 5. The higher the score, the higher the frequency of respondents using this strategy. The Cronbach’s alpha values of these three aspects are 0.75, 0.72, and 0.71.

The nurse–patient relationship was measured using the nurse–patient relationship scale developed by Ma et al. (2020) based on the perspective of nurses, which includes two dimensions and nine items: four items for nurse–patient trust and five items for patient-centered care. Each item was measured on a 6-point Likert scale ranging from 1 to 6. A score of 1 and 6 indicated strong agreement and disagreement, respectively. The lower the score, the worse the nurse–patient relationship. The Cronbach’s alpha value for this scale was noted as 0.90 and 0.88.

Job satisfaction was measured using a questionnaire designed by Pan (2018) based on Herzberg’s two-factor theory, which had a reliability value of 0.84. The questionnaire includes two dimensions and eight items: endogenous and exogenous job satisfaction, each of which contains four items. The questionnaire uses a 5-point Likert scale, expressed on a scale of 1 (*very dissatisfied*) to 5 (*very satisfied*). Higher scores indicate higher job satisfaction.

## 4. Results

### 4.1. Preliminary analysis

This study applied structural equation modeling (SEM) with maximum likelihood estimation (MLE) using AMOS 23.0 (Yuan and Bentler, 2007). This technique provides robust analysis of data, which allows “researchers to simultaneously analyze complex inter-relationships between observed and latent variables” and estimate model fit values (Lai, 2018; Sarstedt et al., 2020; Islam et al., 2022a,b,c). However, before performing AMOS, certain pre-assumptions (e.g., missing values, outliers, normality, and multicollinearity) need to be fulfilled (Finney and DiStefano, 2013). We found that there were no missing values in the data because it was collected through the online platform with the “no skip” option enabled. In addition, using the stem-leaf method, the study found no extreme values. The values of skewness and kurtosis were used to check the normality of the data, with all values within the recommended ranges of ±1 and ± 3, respectively (Byrne, 2010). The correlational values were below the criteria of 0.85 (see Table 1) which indicated absence of multicollinearity. The study further used Harman’s single-factor test for common method bias (CMB) and noted 45.67% variance from a single factor, which met the criterion of less than 50% (Podsakoff et al., 2012).

**Table 1:** Scores, correlations and the square root of AVE among variables.

	M ± SD	1	2	3	4	5	6	7
1. Exogenous job satisfaction	4.13 ± 0.65	**0.88**						
2. Endogenous job satisfaction	4.04 ± 0.73	0.74^**^	**0.89**					
3. Surface acting	3.13 ± 0.96	−0.31^**^	−0.29^**^	**0.82**				
4. Deep acting	3.80 ± 0.73	0.32^**^	0.31^**^	−0.10^*^	**0.83**			
5. Naturally felt emotions	3.52 ± 0.68	0.28^**^	0.25^**^	−0.25^**^	0.59^**^	**0.80**		
6. Nurse–patient trust	4.80 ± 0.78	0.39^**^	0.35^**^	−0.43^**^	0.27^**^	0.37^**^	**0.87**	
7. Patient-centered nursing	5.00 ± 0.73	0.37^**^	0.32^**^	−0.37^**^	0.27^**^	0.35^**^	0.78^**^	**0.84**

^*^p < 0.05, ^**^p < 0.01. The bold numbers represent the square root of AVE.

M, means; SD, standard deviations.

### 4.2. Descriptive and correlation analysis

Table 1 shows the means, standard deviations, and correlation coefficients among our research variables. The values show (Table 1) that respondents agreed regarding exogenous job satisfaction (*M* = 4.13), endogenous job satisfaction (*M* = 4.04), DA (*M* = 3.80), nurse–patient trust (*M* = 4.80) and patient-centered nursing (*M* = 4.75); however, they remained neutral regarding SA (*M* = 3.13) and NFE (*M* = 3.62). The results further showed that the SA was statistically significantly negatively correlated with other variables (*p* < 0.05), and all other variables were statistically significantly positively correlated (*p* < 0.05) (for more details about correlations, see Table 1).

### 4.3. Confirmatory factor analysis

We examined the measurement model using confirmatory factor analysis (CFA) to test the adequacy of the model. We used Cronbach’s alpha coefficient to test the internal consistency of the scale. The Cronbach’s alpha coefficients was above 0.7 for all seven dimensions, indicating that the scale and each dimension in this study have good reliability (Table 2). The factor loadings of all items were above the standard criteria of 0.30 (Table 2). The model fit indicators were: Chi-Square/Degrees of freedom (χ^2^/df) < 3, root-mean-square error of approximation (RMSEA) < 0.8, goodness-of-fit index (GFI) > 0.9, adjusted goodness-of-fit index (AGFI) > 0.9, normed fit index (NFI) > 0.9, comparative Fit Index (CFI) > 0.9, Tucker-Lewis index (TLI) > 0.9 (Byrne, 2010). For convergent validity, the study examined the values of composite reliability (CR ≥ 0.60) and average variance extracted (AVE ≥ 0.50); whereas, for discriminant validity the study examined that whether the square root of AVE was greater than the values of correlation (Hair et al., 2010).

**Table 2:** Factor loading and validity results.

Items	Loading	CR	AVE	√AVE	α
Exogenous job satisfaction	0.85–0.94	0.93	0.78	0.88	0.95
Endogenous job satisfaction	0.87–0.93	0.94	0.79	0.89	0.98
Surface acting	0.73–0.85	0.93	0.67	0.82	0.97
Deep acting	0.79–0.90	0.90	0.69	0.83	0.91
Naturally felt emotions	0.63–0.87	0.84	0.64	0.80	0.96
Nurse–patient trust	0.82–0.95	0.92	0.76	0.87	0.93
Patient-centered nursing	0.77–0.87	0.92	0.71	0.84	0.98

CR, composite reliability; AVE, average variance extracted; √AVE, the square root of AVE, α, the Cronbach’s alpha value.

In our study, the main fit indicators showing acceptably good model fit were as follows: χ^2^/df = 1.973, RMSEA = 0.044, GFI = 0.925, AGFI = 0.904, TLI = 0.984, CFI = 0.986, NFI = 0.973. Furthermore, the values of AVE and CR were above the standard criteria (Table 2), and the values of correlation were noted less than the square root of AVE (Table 1).

### 4.4. Hypotheses testing

**Figure 2:**
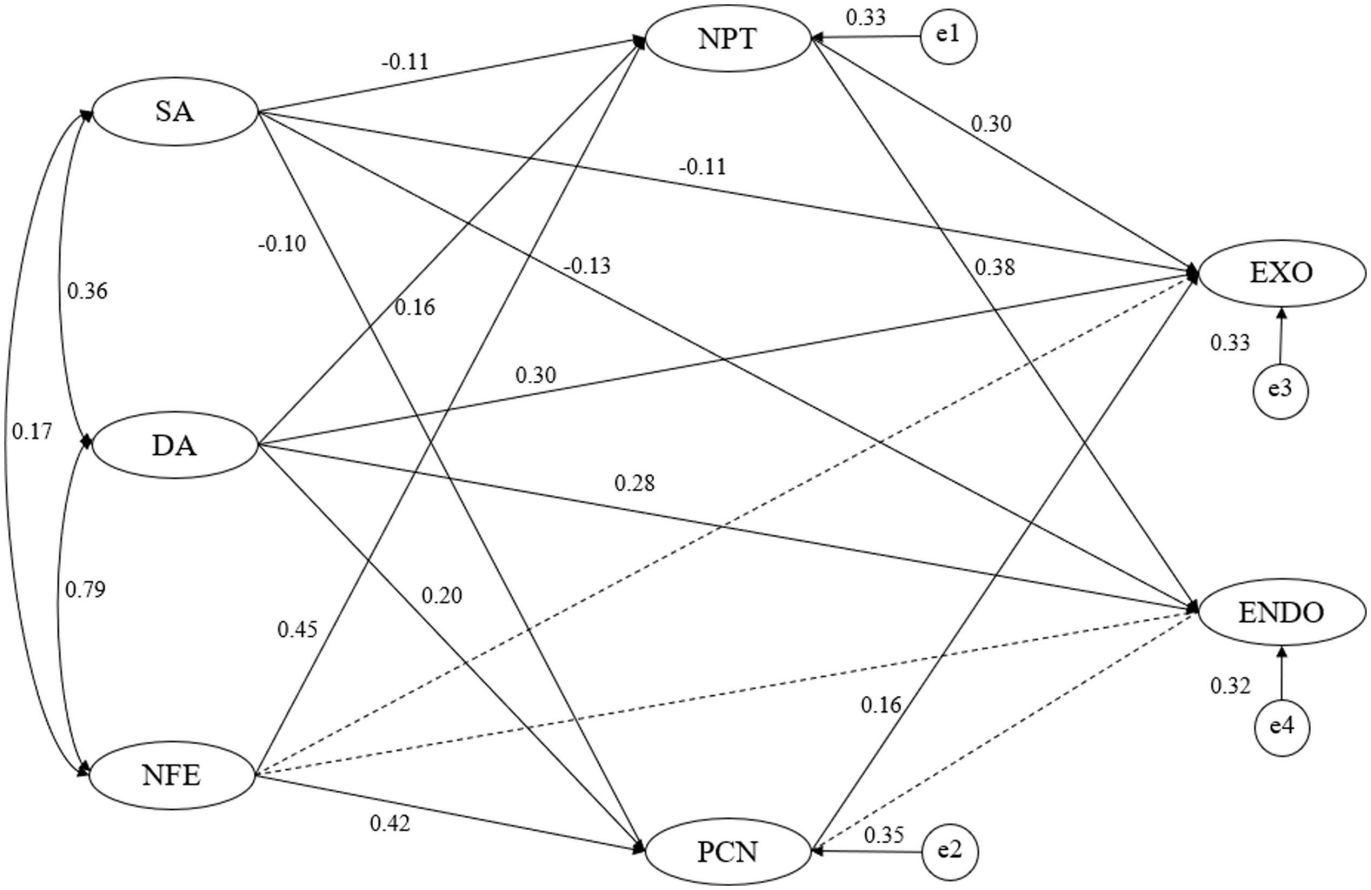
The model with standardized path coefficients. Solid lines represent significant paths, dashed lines represent insignificant paths. e, residual error; ENDO, endogenous job satisfaction; EXO, exogenous job satisfaction; SA, surface acting; DA, deep acting; NFE, naturally felt emotions; NPT, nurse-patient trust; PCN, patient-centered nursing.

To elucidate the relationship between the variables, a path analysis was performed. The results showed that there were no statistically significant differences in the direct effects of “patient-centered care on endogenous job satisfaction” and “NFE on endogenous and exogenous job satisfaction” (*p* > 0.05). The remaining paths were statistically different (*p* < 0.05), indicating that the assumptions held (Table 3; Figure 2).

**Table 3:** Path analysis results.

Hypothesized relationship	β	SE	*t*-value	*p*	Results
SA → NPT	−0.109	0.033	−2.544	0.011	Supported
SA → PCN	−0.101	0.029	−2.343	0.019	Supported
DA → NPT	0.160	0.091	2.043	0.041	Supported
DA → PCN	0.198	0.082	2.513	0.012	Supported
NFE → PCN	0.415	0.070	5.779	***	Supported
NFE → NPT	0.450	0.078	6.245	***	Supported
SA → EXO	−0.113	0.026	−2.525	0.012	Supported
NPT → EXO	0.295	0.060	3.763	***	Supported
SA → ENDO	−0.128	0.031	−2.983	0.003	Supported
PCN → ENDO	0.086	0.078	1.153	0.249	Denied
DA → EXO	0.304	0.073	3.693	***	Supported
DA → ENDO	0.284	0.086	3.605	***	Supported
NFE → ENDO	0.061	0.075	0.832	0.406	Denied
NFE → EXO	0.075	0.064	0.983	0.325	Denied
NPT → ENDO	0.381	0.071	5.088	***	Supported
PCN → EXO	0.158	0.066	2.037	0.042	Supported

^***^p < 0.001.

β, standardized path coefficient; SE, standard error; ENDO, endogenous job satisfaction; EXO, exogenous job satisfaction; SA, surface acting; DA, deep acting; NFE, naturally felt emotions; NPT, nurse–patient trust; PCN, patient-centered nursing.

### 4.5. Mediation analysis

Using the bias-corrected percentile Bootstrap method to test the mediation effect, we set the bootstrap sample size to 5,000 and the confidence interval level to 95%. If the 95% confidence intervals for the indirect effect did not contain zero, it indicated a statistically significant mediating effect (Hayes, 2009). In order to show the mediating effect more clearly, we classified different strategies of EL and tested them individually according to the above-mentioned assumptions that have been verified.

The results showed that the mediating effects of nurse–patient trust and patient-centered nursing on SA and exogenous job satisfaction were both established, and the mediating effect of nurse–patient trust on SA and endogenous job satisfaction was also established (Table 4A). Likewise, the nurse–patient relationship (nurse–patient trust and patient-centered care) partially mediated the effects of EL (DA and NFE) on job satisfaction (exogenous and endogenous) (Tables 4B, 4C).

**Table 4A:** Mediating effect of nurse–patient relationship between the surface acting and job satisfaction.

	Effect	Coefficient	SE	LLCI	ULCI
SA → NPT → EXO	Total Indirect	−0.096	0.025	−0.153	−0.055
SA → PCN → EXO					
SA → EXO	Direct	−0.109	0.036	−0.177	−0.035
SA → EXO	Total	−0.205	0.024	−0.253	−0.158
SA → NPT → ENDO	Indirect	−0.089	0.025	−0.145	−0.045
SA → ENDO	Direct	−0.132	0.041	−0.209	−0.052
SA → ENDO	Total	−0.221	0.030	−0.280	−0.163

LLCI, lower level of confidence interval; SE, standard error; ULCI, upper level of confidence interval; ENDO, endogenous job satisfaction; EXO, exogenous job satisfaction; SA, surface acting; NPT, nurse–patient trust; PCN, patient-centered nursing.

**Table 4B:** Mediating effect of nurse–patient relationship between the deep acting and job satisfaction.

	Effect	Coefficient	SE	LLCI	ULCI
DA → NPT → EXO	Total Indirect	0.085	0.023	0.045	0.135
DA → PCN → EXO					
DA → EXO	Direct	0.196	0.046	0.106	0.290
DA → EXO	Total	0.282	0.047	0.194	0.377
DA → NPT → ENDO	Indirect	0.076	0.022	0.038	0.124
DA → ENDO	Direct	0.229	0.051	0.129	0.331
DA → ENDO	Total	0.306	0.050	0.209	0.407

LLCI, lower level of confidence interval; SE, standard error; ULCI, upper level of confidence interval; ENDO, endogenous job satisfaction; EXO, exogenous job satisfaction; DA, deep acting; NPT, nurse–patient trust; PCN, patient-centered nursing.

**Table 4C:** Mediating effect of nurse–patient relationship between the naturally felt emotions and job satisfaction.

	Effect	Coefficient	SE	LLCI	ULCI
NFE → NPT → EXO	Total Indirect	0.127	0.030	0.074	0.191
NFE → PCN → EXO					
NFE → EXO	Total	0.269	0.053	0.172	0.379
NFE → NPT → ENDO	Indirect	0.119	0.030	0.066	0.183
NFE → ENDO	Total	0.265	0.054	0.161	0.372

LLCI, lower level of confidence interval; SE, standard error; ULCI, upper level of confidence interval; ENDO, endogenous job satisfaction; EXO, exogenous job satisfaction; NFE, naturally felt emotions; NPT, nurse–patient trust; PCN, patient-centered nursing.

## 5. Discussion

This study used structural equation modeling to illustrate the relationship between EL, nurse–patient relationships, and job satisfaction. In addition, the Bootstrap procedure was used to test mediating effects of nurse–patient relationship (nurse–patient trust and patient-centered care).

The results showed that SA and DA and negatively and positively predicted nurse–patient relationship and satisfaction, respectively, which was consistent with previous research findings (Hülsheger and Schewe, 2011; Wu et al., 2018; Yeh et al., 2020). Nurses have different emotional roles in caring for patients. When they hold two psychologically inconsistent cognitions at the same time, they experience a state of negative drive called dissonance that can lead to lack of self-esteem, depression, cynicism, and alienation from work (Blake and Ronald, 1993). If real emotions are expressed or the cognitive understanding of emotional events is changed so that one’s true feelings are in line with the emotional expressions expected by the organization, the emotional resources will continue to increase, and the satisfaction will be improved (Alvaro et al., 2010; Shao et al., 2016).

In terms of predicting job satisfaction, DA is more likely to restore positive states and increase job satisfaction. By evaluating Chinese nurses, Wu et al. (2018) also concluded that increasing the ability and frequency of DA may improve nurses’ job satisfaction. Deeper actions can change one’s innermost feelings to match organizational expectations and produce more natural and authentic emotional expressions (Alicia, 2003). At the same time, the fact that the highest scores in the field of DA of EL suggest that nurses may work to overcome this emotional challenge with a strong sense of responsibility and commitment to show themselves the emotional attitudes their roles should have (Kim and Jang, 2018). Therefore, it is essential to develop an EL strategy of DA that would help them maintain this edge. Although the direct effect of NFE on job satisfaction was not significant in the model, this was inconsistent with the results of other researchers (Shao et al., 2016; Yin and Wang, 2018), which may be due to differences in study populations and organizational cultures. The department in which nurses work, the level of stress placed on them and the duration of their relationship with patients are of great importance and may affect the results. For example, in the emergency department, where a strong patient–nurse relationship is not formed and the communication period is short, maybe NFE which was not significant in this study, becomes doubly important because the patients in this department experience intense negative emotions at the moment. Maybe a natural and real excitement in the nurse of this department can be very effective in the formation of the relationship and on the other hand positive feedback from the environment and bring higher job satisfaction (Carter et al., 2014; Mirzaei et al., 2022). Furthermore, for a nurse who works in the psychiatric department of a hospital and is faced with an exaggerated flow of extreme positive and negative emotions of patients every day, SA may be a more suitable emotional reaction and bring more job satisfaction (Cramer et al., 2020; Young and Hee, 2022).

However, we found that NFE could affect job satisfaction through an indirect effect of the nurse–patient relationship. This suggests that nurses could improve satisfaction by changing their relationship with patients through this EL strategy. Cheung et al. (2018) pointed out that if employees combined DA with NFE, their occupational wellbeing improved and that understanding or using an EL strategy was not sufficient.

This study also verified a series of mediating effects of the nurse–patient relationship on different emotional strategies and job satisfaction. Specifically, both nurse–patient trust and patient-centered nursing mediated different strategies of EL and exogenous job satisfaction. Only nurse–patient trust mediated different strategies of EL and endogenous job satisfaction. The nurse–patient relationship is a kind of interpersonal relationship that belongs to the external level, and the nurse–patient relationship is largely influenced by the external environment (Ma et al., 2020). This may explain why the mediating effect of the nurse–patient relationship is more accounted for in EL and exogenous job satisfaction. Research showed that emotional management enabled nurses to better use their emotions during patient treatment to have a higher quality nurse–patient relationship, improve patient safety, and ensure higher nurse and patient satisfaction (Kim and Jang, 2018; Vujanić et al., 2020). Appropriate EL positively affects patient relationships and raises patient trust in nurses’ motivations and behaviors (Hong and Kim, 2018). However, long-term inconsistency between one’s own experience and the professional mood required by the hospital can lead to emotional dysregulation in the nursing staff (Back et al., 2017), and negative nurse–patient interaction reduces job satisfaction. Therefore, nurses need to constantly adjust their emotions according to the situation to ensure a positive interaction with patients. Interestingly, nurse–patient trust, as one of the parallel mediators in this study, had greater predictive value and impact. Nursing-patient trust is the cornerstone of the nurse–patient relationship, and a social atmosphere of cooperation and trust among nurses improves the job quality of patient care (McNeil et al., 2019). In other words, enhancing nurse–patient trust is a more valuable aspect for nurses who may have low levels of job satisfaction.

### 5.1. Theoretical implications

This study has made significant contributions in several aspects. First, although a large number of studies have reported the relationship between EL and job satisfaction (Back et al., 2017; Wu et al., 2018; Young and Hee, 2022), the mechanism of how EL affects job satisfaction has not been fully explored, especially in nursing. This study indicated that the relationship between nurses’ EL and job satisfaction could be explained by the nurse–patient relationship, especially the nurse–patient trust. These findings emphasize the importance of nursing-patient trust as mediator in the effect of EL on job satisfaction and help researchers better grasp the internal mechanisms. Second, previous studies have shown inconsistencies in the relationship between EL and job satisfaction (Shao et al., 2016; Yin and Wang, 2018). Our study explored the relationship in detail and highlights the positive EL that can benefit nurses personally and their work. Finally, this study contributed to the existing literature on how EL affects job satisfaction through nurse–patient relationship which is rare in literature, and provided evidence for improving satisfaction through more effective EL and good nurse–patient relationship.

### 5.2. Practical implications

EL is considered an important part of the role of the health care professional, especially in the nursing field (Mann and Mark, 2005; Zamanzadeh et al., 2013). Nursing is not only task-based but also emotion-based work (Kim, 2018). Emotion management allows nurses to engage with patients on a more personal level, which is thought to be a particularly satisfying part of their job role (Mann and Mark, 2005). Therefore, the aim of nursing managers in respect of EL must be to attempt to reduce its negative consequences whilst retaining the positive outcomes for both patient and nurses. Strategies can be employed to counteract the negative effects of EL performance and promote conversion to DA and NFE. In addition, more opportunities should be provided to discuss the use of different strategies for EL and encourage appropriate responses based on the patient’s situation and work context (Ding et al., 2019; Kim, 2020).

Our study indicated that nurse–patient relationship played a key role in mediating the effect of EL on endogenous and exogenous satisfaction to a job. Due attention should be paid to how to develops nurse–patient relationships, especially nurse–patient trust, which will affect job satisfaction. Specifically, nurses’ professional competence and interpersonal caring traits become the most important factors in developing trust. Studies found that it was possible to work with patients and their families. For example, in developing patient-centered practices and holistic care plans, information sharing can be increased, and more democratic partnerships can be established (Gou et al., 2021a,b). Moreover, nurse–patient trust is largely affected by the social and hospital organizational environments (Ma et al., 2020). In groups with a higher level of organizational citizenship behavior, whether due to personal motivation or with the help of others, nurses are more likely to perform better clinical care of patients, resulting in improved relationships between nurses and patients (Smith and Lorentzon, 2005; Zamperini et al., 2015; Gou et al., 2021a,b). This also prompts the importance of creating a good environment and atmosphere for nurses to work. Nursing managers should continuously evaluate and examine the trust phenomenon in the nurse–patient relationship and take corresponding measures to improve the awareness of clinical nurses.

### 5.3. Limitations

This study is a cross-sectional survey. Although, according to literature and theories, causal relationships between correlated variables were verified using structural equation modeling, this study only measured these variables at a certain time point. Longitudinal studies can be carried out in the future to observe dynamic changes in the relationship between nurses’ EL, nurse–patient relationship, and job satisfaction. Second, the sample selection for this study was limited, which limits the generalization of the research results to a certain extent. In future research, we can expand the area to conduct a large sample survey of nurses in different places to enrich the representativeness of the sample. Third, this study only explored the mediating effect of the nurse–patient relationship between EL and job satisfaction. Since there are many influencing factors of variables (such as personal traits, organizational support, and work environment, etc.), future research can analyze data at different levels to supplement more detailed results. In addition, the nurse–patient relationship in this study was self-reported based on a nurse’s perspective. Future studies could incorporate patient evaluations to make the results more objective and complete.

## 6. Conclusion

Nurses’ SA negatively predicted nurse–patient relationship and job satisfaction, whereas DA and natural felt emotions positively predicted nurse–patient relationship and job satisfaction. The nurse–patient relationship plays a mediating role in nurses’ EL and job satisfaction, and nurse–patient trust plays an important pant. Targeted interventions should be developed and implemented to mitigate the negative effects of EL in nurses, promote the use of deep behaviors, increase focus on nurse–patient trust, and improve satisfaction and quality of care.

Nurses should continuously adjust their EL strategies according to the situation, reduce SA, increase DA and NFE, increase the positive interaction and trust between nurses and patients, and improve job satisfaction. Nurse researchers can use these findings as a reference to develop interventions. Nursing managers should pay more attention to the EL of nurses, bringing into play their positive EL and reducing their emotional exhaustion. Nursing managers should take measures to improve the nurse–patient relationship and create a favorable environment and atmosphere for nurses’ nursing work.

## Data availability statement

The original contributions presented in the study are included in the article/supplementary material, further inquiries can be directed to the corresponding author.

## Ethics statement

The studies involving human participants were reviewed and approved by the Ethics Committee of Shengjing Hospital of China Medical University (2018PS09K). The patients/participants provided their written informed consent to participate in this study.

## Author contributions

Y-wX made substantial contributions to conception and design, acquisition of data, and analysis and interpretation of data. Y-wX and LF involved in drafting the manuscript and revising it critically for important intellectual content. All author participated sufficiently in the work to take public responsibility for appropriate portions of the content and agreed to be accountable for all aspects of the work in ensuring that questions related to the accuracy and integrity of any part of the work are appropriately investigated and resolved.

## Funding

This project was funded by the Department of Science and Technology of Liaoning Province (grant no. 2018225005).

## Conflict of interest

The authors declare that the research was conducted in the absence of any commercial or financial relationships that could be construed as a potential conflict of interest.

## Publisher’s note

All claims expressed in this article are solely those of the authors and do not necessarily represent those of their affiliated organizations, or those of the publisher, the editors and the reviewers. Any product that may be evaluated in this article, or claim that may be made by its manufacturer, is not guaranteed or endorsed by the publisher.
